# Genomic Analysis Reveals Pleiotropic Alleles at EDN3 and BMP7 Involved in Chicken Comb Color and Egg Production

**DOI:** 10.3389/fgene.2019.00612

**Published:** 2019-06-28

**Authors:** Xianggui Dong, Junying Li, Yuanyuan Zhang, Deping Han, Guoying Hua, Jiankui Wang, Xuemei Deng, Changxin Wu

**Affiliations:** ^1^National Engineering Laboratory for Animal Breeding and Key Laboratory of Animal Genetics, Breeding, and Reproduction of the Ministry of Agriculture, China Agricultural University, Beijing, China; ^2^College of Veterinary Medicine, China Agricultural University, Beijing, China

**Keywords:** chicken, comb color, egg production, pleiotropy, signatures of selection

## Abstract

Artificial selection is often associated with numerous changes in seemingly unrelated phenotypic traits. The genetic mechanisms of correlated phenotypes probably involve pleiotropy or linkage of genes related to such phenotypes. Dongxiang blue-shelled chicken, an indigenous chicken breed of China, has segregated significantly for the dermal hyperpigmentation phenotype. Two lines of the chicken have been divergently selected with respect to comb color for over 20 generations. The red comb line chicken produces significantly higher number of eggs than the dark comb line chicken. The objective of this study was to explore potential mechanisms involved in the relationship between comb color and egg production among chickens. Based on the genome-wide association study results, we identified a genomic region on chromosome 20 involving *EDN3* and *BMP7*, which is associated with hyperpigmentation of chicken comb. Further analyses by selection signatures in the two divergent lines revealed that several candidate genes, including *EDN3*, *BMP7*, *BPIFB3*, and *PCK1*, closely located on chromosome 20 are involved in the development of neural crest cell and reproductive system. The two genes *EDN3* and *BMP7* have known roles in regulating both ovarian function and melanogenesis, indicating the pleiotropic effect on hyperpigmentation and egg production in blue-shelled chickens. Association analysis for egg production confirmed the pleiotropic effect of selected loci identified by selection signatures. The study provides insights into phenotypic evolution due to genetic variation across the genome. The information might be useful in the current breeding efforts to develop improved breeds for egg production.

## Introduction

Domestication is the strongest type of directional selection, resulting in the accumulation of numerous phenotypic variations in animals. During the last two decades, numerous genetic bases of traits accounting for the causative mutation of interesting phenotypes in domestic animals have been deciphered (Andersson and Georges, 2004). In livestock, it has been reported that vibrant appearance not only represents the physiognomy but also relates to development, health, and production traits. Such changes in unrelated phenotypes are potentially caused by pleiotropy or linkage between independent genetic architectures (Wright et al., 2010). Pleiotropy refers to a phenomenon in which a single gene or genetic variant affects multiple apparently unrelated phenotypic traits (Stearns, 2010). Several types of pleiotropy, including biological pleiotropy and spurious pleiotropy, have been defined (Hodgkin, 1998; Solovieff et al., 2013). Biological pleiotropy is described as a gene or genetic variant having a direct biological effect on more than one phenotypic trait, and the different causal variants are tagged by the same gene or genetic variant. Spurious pleiotropy is where the causal variants in different genes are in strong linkage disequilibrium (Hodgkin, 1998; Solovieff et al., 2013). Genome-wide association studies (GWASs) on human complex diseases (Solovieff et al., 2013; Pickrell et al., 2016) and livestock traits have revealed that pleiotropy is common in vertebrates (Rosengren Pielberg et al., 2008; Johnsson et al., 2012; Curik et al., 2013; Johnsson et al., 2014).

The comb of birds is a sexual ornament that is used as an indicator of sexual maturity and also related to mate choice in wild populations (Mukhtar and Khan, 2012). In female chicken, the size of comb is correlated to fecundity; for instance, domestic layers with large combs produce more eggs, which is potentially regulated by hormones (Wright et al., 2012), whereas, in male chicken, the comb size is inversely correlated to sperm viability (Navara et al., 2012). The type of comb is associated with sperm quality; for example, roosters with rose combs carrying homozygous allele have defective sperm motility (Crawford and Smyth, 1964; Imsland et al., 2012). The color of comb is also linked with sperm viability; for instance, it was reported that males with redder combs have higher percent of active sperms (Navara et al., 2012). In general, combs are colored red, yellow, or orange due to the blood engorged in the superficial dermis, which is richly vascularized, and deposition of carotenoid pigments in the epidermis (Stettenheim, 2000). However, several breeds, for instance, the well-known silky chicken, present dark or black combs owing to the heavy dermal melanization of the internal tissue, which is known as fibromelanosis (FM). Silky chicken is an extensively studied breed displaying FM. It has been reported that FM is caused by inverted duplication and combination of two genomic duplications separated by a >400-Kb region on chromosome 20. The endothelin 3 (*EDN3*) gene, which encodes a potent mitogen in melanocytes, is involved in the first duplication and is considered the causal gene of FM (Dorshorst et al., 2011; Shinomiya et al., 2012).

**Figure 1:**
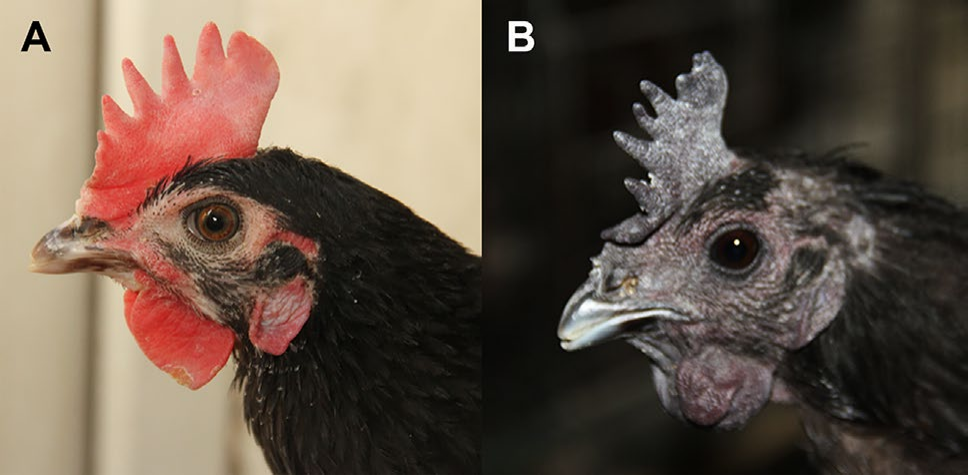
Comb color phenotype in Dongxiang blue-shelled chicken. **(A)** Chicken comb with typical red color. **(B)** Dark chicken comb, the wattle, face, and ear lobe also present fibromelanosis phenotype.

China has a wide range of chicken breeds with various phenotypes. Dongxiang blue-shelled chicken (*Gallus gallus*) is a specific indigenous breed that originated from Jiangxi Province, South China. Blue egg is the main characteristic of Dongxiang blue-shelled chicken, and the associated gene has been fixed, and the genetic mechanism of blue eggshell color has been deciphered (Wang et al., 2013). Besides the blue eggshell trait, another dermal hyperpigmentation trait has also been segregated in Dongxiang blue-shelled chicken population. There are two different comb colors in the chicken, namely, dark and red (**Figure 1**). Almost all the individuals with dark comb present a unique collection of dark phenotypes such as dark wattle, face, beak, skin, and muscle, whereas the red comb chickens have white or normal colored organs. The visible dark phenotypes in livestock are due to intense pigmentation of the dermal layer of the skin. Based on the comb color, two chicken lines were generated for over 20 generations since the early 2000, namely, dark comb line (DCL) and red comb line (RCL). Simultaneously, a mixed population was kept containing both red comb chickens and dark comb chickens and mated randomly during breeding, resulting in the random line (RAL). Except for the comb appearance, there are no obvious differences between the two chicken lines. As mentioned, the RCL has higher egg production than that of the DCL. Such a difference is found not only in just one breed, but in another Chinese local breed, Dehua black chicken also has two types of comb color. Egg production in red comb chickens is approximately 25% higher than that in dark comb chickens (Hongtao, 2006).

The study suggests that dark comb phenotype is associated with a high incidence of low egg production in chicken. Moreover, we speculate that a potential genetic pleiotropism might link the comb color and egg production. Therefore, in this study, we aimed to explore potential mechanisms involved in the relationship between comb color and egg production among chickens.

## Materials and Methods

### Ethical Statement

Regular quarantine inspection of the experimental farm in China Agricultural University was conducted. The blood samples were collected from brachial veins of chickens by standard venipuncture. The whole procedure was carried out strictly following the protocol approved by the Animal Care Committee of China Agricultural University.

### Animals and Phenotype

The samples utilized in the study were from three lines of chicken in the experimental farm of China Agricultural University. A total of 178 female chickens were obtained from the following three lines: 86 chickens were from RCL, 30 chickens from DCL, and 62 chickens from RAL. In the RAL, 14 were red comb chickens and 48 were dark comb chickens (**Table 1**). The number of eggs from weeks 17 to 60 was recorded for these chickens. The number of eggs was also recorded in another large population (*N* = ∼500) from a conservation farm of Dongxiang blue-shelled chicken. Frozen section of comb tissues (*N* = 6 for DCL and RCL) were prepared and stained with hematoxylin and eosin following the protocol of a previous study (Han et al., 2015). The blood parameters were determined using an automated hematology analyzer and the ferroheme kit (Sino-uk Institute, Beijing, China).

**Table 1:** Description of the samples used in the genomic analyses.

Population	RCL	DCL	RAL		Total
Comb color	Red	Dark	Red	Dark	
Prior to QC	86	30	14	48	178
After QC	86	29	14	44	173
Dataset1			14	44	58
Dataset2	86	29			115

### Genotyping and Quality Control

The genomic DNA was extracted from blood samples using the avian blood DNA extraction kit (Tiangen Co., LTD., Beijing, China). Genotyping was carried out using the Affymetrix Axiom 600K Chicken Genotyping Array (Affymetrix, Inc., Santa Clara, CA, USA) with a total of 580,691 single nucleotide polymorphisms (SNPs) (Kranis et al., 2013); all genome coordinates are representative of the *Gallus_gallus-5.0* reference assembly. The first-step quality control and genotype calling from the raw data in the form of CEL files were performed using Affymetrix Power Tools v1.16.0 software with the criterion of dish quality control of >0.82 and call rate of >97%. The second-step quality control was then implemented with plink v1.9 software (Purcell et al., 2007). The SNPs with unknown genomic positions and redundant coordinates were removed. The SNPs with a call rate of <90% and a minor allele frequency of <5% were excluded. The SNPs that deviated from Hardy–Weinberg equilibrium (*P* < 1E-6) were removed. Five individuals (one in DCL and four in RAL) with a call rate of <90% were removed. Finally, 173 chickens and 387,704 SNPs remained for the further analysis.

### Analysis of Population Structure

Prior to the population structure analysis, all autosomal SNPs were pruned using the indep-pairwise option with a window size of 25 SNPs, step of five SNPs, and *r* ^2^ threshold of 0.2. After pruning, 36,853 independent SNPs remained. The principal component analysis (PCA) was carried out using GCTA software (Yang et al., 2011) to assess population structure using these independent SNPs.

### Genome Wide Association Analysis

We conducted an association analysis of comb color phenotype with genotypic polymorphism using a univariate linear mixed model in Gemma software (Zhou and Stephens, 2012). Simultaneously, potential cryptic relatedness and hidden population stratification were corrected in the model. We estimated the centered relatedness matrix using independent SNPs implemented in Gemma software prior to the association analysis. The univariate linear mixed model for each SNP marker is as follows:

y=Wα+xβ+u+ε

where, *y* is an *n*-vector of binary comb color trait for all RAL individuals; *W* is an *n* × *c* matrix of covariates (fixed effects) including a column of 1s; α is a *c*-vector of the corresponding coefficients including the intercept; *x* is an *n*-vector of SNPs genotypes tested in association analysis, β is the effect size of the SNP; and *u* is an *n*-vector of random effects with a covariance structure as *u* ∼ MVN*_n_* (0; λτ ^−1^ *K*) (where τ ^−1^ is the variance of the residual errors, λ is the ratio between the two variance components, and *K* is a known *n* × *n* relatedness matrix). MVN*_n_* denotes the *n*-dimensional multivariate normal distribution. ε is an *n*-vector of errors. We used Wald test statistic for each SNP testing.

### Analysis of Signatures of Selection

Red comb chickens from RCL and dark comb chickens from DCL (**Table 1**, Dataset2) were used to identify signatures of selection due to divergent selection. Two independent approaches, viz., Weir and Cockerham’s F_ST_ (Weir and Cockerham, 1984) and cross-population extended haplotype homozygosity (XP-EHH) (Sabeti et al., 2007), were implemented in the study. We scanned the whole genome to identify regions with increased genetic divergence (F_ST_) between chickens with red and dark combs. We used VCFtools (v0.1.13) (Danecek et al., 2011) to calculate F_ST_ and performed Z transformation. The genomic regions with a high ZF_ST_ (1% quantiles) were analyzed for gene contents. The statistic of XP-EHH calculated for each SNP in the dataset was implemented in Selscan v-1.1.0b (Szpiech and Hernandez, 2014). The XP-EHH statistic was also transformed to Z distribution. Prior to the XP-EHH test, fastPHASE v1.4 software (Scheet and Stephens, 2006) was implemented to estimate missing genotypes and reconstructing haplotypes from unphased SNP genotype data. For DCL–RCL comparison, the XP-EHH results were standardized for each chromosome separately, and the 1% quantiles of the standardized XP-EHH distribution for all SNPs were used as threshold to identify outlier SNPs.

### Gene Enrichment Analysis

The gene contents in the candidate regions were annotated according to the outliers tested by XP-EHH and F_ST_ from the University of California—Santa Cruz database (galGal5). Firstly, we converted the chicken Ensembl transcript IDs to human orthologue gene IDs from Ensembl Gene 93 Database using the online tool BioMart (Kinsella et al., 2011). The Gene Ontology (GO) and Kyoto Encyclopedia of Genes and Genomes pathway analyses were performed in the blue-shelled chickens with Metascape (http://metascape.org). Benjamini–Hochberg false discovery rate was used for correcting the *P* values. The candidate genes were classified into categories: molecular function, biological process (BP), and cellular component.

### Association Analysis With Selected Single Nucleotide Polymorphism

The association analysis between the SNPs potentially under positive selection and the number of eggs from weeks 17 to 60 was carried out with the function –*assoc* in PLINK software (Purcell et al., 2007).

### Verification of Duplications in Dark Comb Chickens

To test the boundaries of the two duplications causing the FM phenotype, a three primer diagnostic test for the presence of each of the duplications (Dorshorst et al., 2011) was used, following a touchdown thermal cycling protocol: 95°C for 5 min, 16 cycles of 95, 68 (−1°C/cycle), and 72°C for 30 s each, followed by 24 cycles of 95, 52, and 72°C for 30 s each.

### Candidate Gene Expression

The total RNA was extracted from the ovary using Trizol reagent, followed by synthesis of cDNA from 1 μg of RNA using the reverse transcription kit (Tiangen Co., LTD., Beijing, China). Four pairs of primers designed for the four candidate genes (*EDN3*, *BMP7*, *BPIFB3*, and *PCK1*) and the housekeeping gene *GAPDH* are presented in **Supplementary Table 1**; all primers span one intron at least. The quantitative real-time polymerase chain reaction (qPCR) was performed for the four candidate genes from the DCL (*N* = 6) and RCL (*N* = 6) chickens. For the qPCR runs, the target genes and housekeeping gene were performed in three technical replicates, and the *GAPDH* gene was used as the internal control for normalizing the level of expression of the four target genes. The average relative quantitative values were calculated, and the differences in expression levels were analyzed by Student’s *t*-test between DCL and RCL.

## Results

### Difference in Egg Production Between Red Comb and Dark Comb Chickens

**Figure 2:**
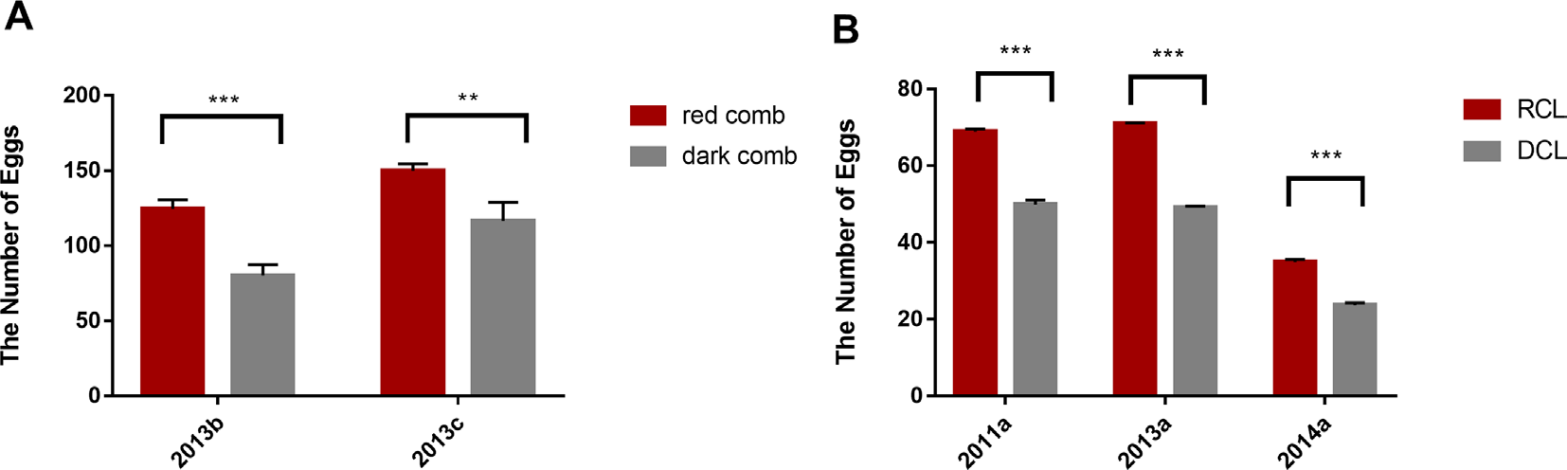
Difference in egg production between dark comb and red comb chickens during laying period. **(A)** Difference in the number of eggs recorded from weeks 17 to 60 obtained from an experimental farm in the China Agricultural University; The coordinate 2013b indicates the comparison between dark comb line (DCL) (*N* = 30) and red comb line (RCL) (*N* = 86); The coordinate 2013c indicates the comparison between dark comb layers (*N* = 48) and red comb layers (*N* = 14) from the random line (RAL). **(B)** Difference in the number of eggs in a large population (*N* = ∼500) in different generations (data obtained from the conservation farm of Dongxiang blue-shelled chicken); the number of eggs were recorded for 4 months (2011a, 2013a) and 2 months (2014a). The coordinates 2011a, 2013a, and 2014a indicate different generations from the conservation farm. ***p < 0.001, **p < 0.01.

We calculated the number of eggs of different populations during the laying period in several generations. The results revealed that the number of eggs produced by RCL was significantly higher than that by DCL, almost throughout the laying period (*P* < 0.001) (**Figure 2A**). The number of eggs produced by the red comb chickens was significantly higher than that by the dark comb chickens in the RAL (*P* < 0.01) (**Figure 2A**). In another large population from the conservation farm of Dongxiang blue-shelled chicken, the number of eggs produced by RCL was significantly higher than that by DCL (*P* < 0.001) (**Figure 2B**).

### Dark Color of Chicken Comb Is Caused by Melanin Pigmentation

We determined the blood parameters, including the level of hemoglobin, heme, and iron (Fe) ion in the red comb and dark comb chickens. No significant difference was observed between the two chicken lines with respect to the blood parameters (**Supplementary Figure 1**). Furthermore, we prepared histologic section of the comb tissue obtained from the two chicken lines. We observed that melanin was deposited in the dark comb tissue and absent in the red comb tissue (**Supplementary Figure 2**). Our findings suggest that melanin instead of blood elements imparts the dark color of comb in chickens.

### Population Structure Analysis

**Figure 3:**
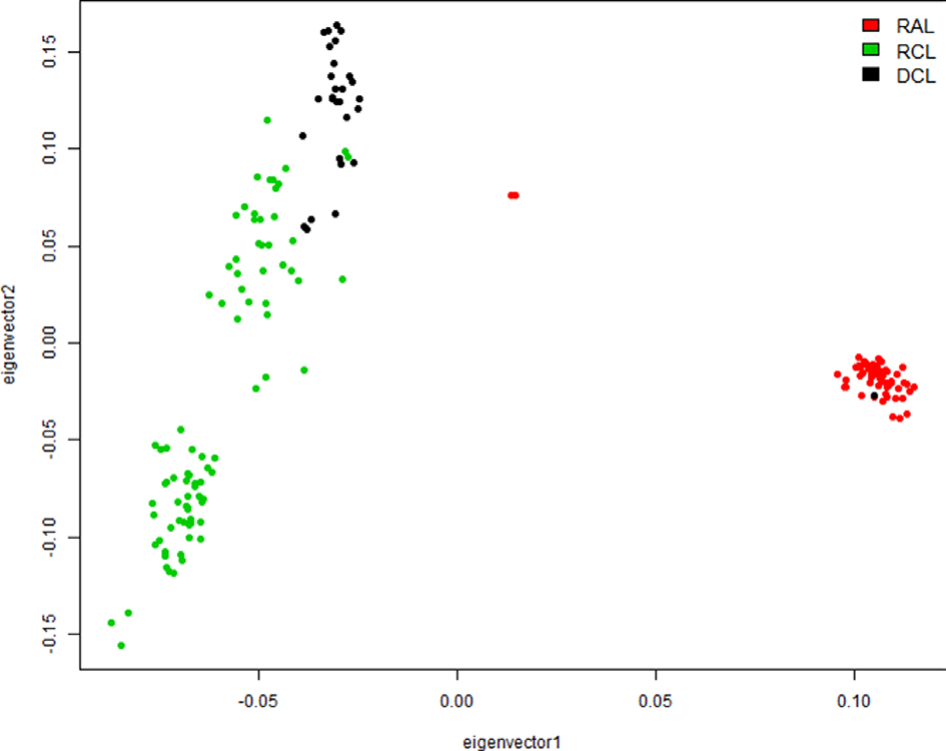
Population structure identified by the principal component analysis (PCA). DCL, RAL, and RCL represent dark comb line, random line, and red comb line chickens, respectively.

The PCA of 36,853 independent SNPs with *r* ^2^ < 0.2 using the first two principal components (**Figure 3**) showed that the three lines formed separate clusters and were well separated from each other. The samples from the RAL were closely clustered except for two outliers and one individual from DCL, probably due to the phenotype recording error, whereas the samples from RCL and DCL were slightly separated from each other, and both of them were far away from the RAL probably because of divergent selection for over 20 generations.

### Identification of Genes Related to Comb Color by GWAS

**Figure 4:**
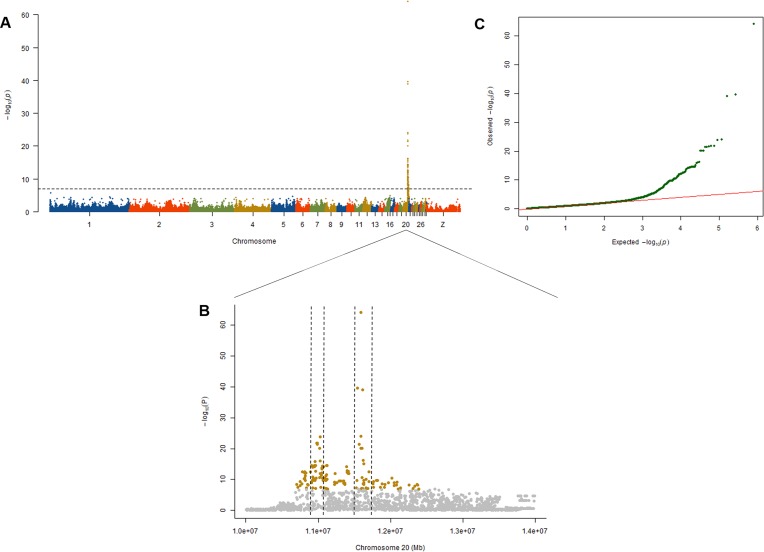
Results from the genome-wide association study (GWAS) of comb color phenotype. **(A)** Manhattan plot of the GWAS for the comb color phenotype; the *y* axis shows −log10 *P* values adjusted by Bonferroni approach for the association tests, and the dashed horizontal line indicates the genome-wide significant threshold value (1.23E-7). **(B)** A scatter plot presenting all the single-nucleotide polymorphisms (SNPs) tested on chromosome 20 for the comb color phenotype. The extremely significant SNPs in the two peaks are in the two duplications related to fibromelanosis (FM). Among these SNPs, 25 are located in duplication 1, and 23 are located in duplication 2. **(C)** The quantile–quantile plot of the *P* values, the *x*-axis shows the expected −log10-transformed *P* values, and the *y*-axis shows the observed −log10-transformed *P* values.

To explore the genetic mechanism of comb color in Dongxiang blue-shelled chickens, we conducted a GWAS by the univariate method for comb color phenotype. As the PCA plot showed that there was a clear differentiation in the three lines (**Figure 3**), we selected 58 chickens from the RAL (**Table 1**, Dataset1), which were clustered together to perform the GWAS of comb color phenotype to reduce the false positive rate of association analysis induced by population stratification. A total of 138 genome-wide significant SNPs (Bonferroni adjusted) located on chromosome 20 were successfully identified for the trait (**Figure 4**, **Table 2**). The association SNPs located between positions 10,705,665 and 12,394,530 were entirely attributable to a chromosomal region (∼1.7 Mb) harboring 24 genes including *EDN3* and bone morphogenetic protein 7 (*BMP7*) (**Supplementary Table 2**). The most significant SNP AX-76199743 (Affymetrix Probe ID) is at position 10,971,168 on chromosome 20. This SNP and another seven SNPs with extremely low *P* values are close to *EDN3* (**Table 2**).

**Table 2:** Significant single nucleotide polymorphisms (SNPs) with extremely high *P* values revealed by genome-wide association studies (GWAS) of comb color phenotype.

Chr.	Affymetrix probe	Position	Allele1	Allele0	*P* value	Duplication	Gene symbol	SNP–gene relationship	Distance (bp)
20	AX-76199743	10971168	C	T	0	Dup 1	EDN3	Downstream	30386
20	AX-76201423	11591137	G	A	8.66E-65	Dup 2	C20H20ORF85	Upstream	78500
20	AX-76201300	11543951	A	G	2.38E-40	Dup 2	C20H20ORF85	Upstream	31314
20	AX-76201480	11616481	A	G	1.08E-39	Dup 2	C20H20ORF85	Upstream	103844
20	AX-76201429	11594767	T	C	8.37E-25	Dup 2	C20H20ORF85	Upstream	82130
20	AX-76199919	11030966	C	T	1.80E-24	Dup 1	EDN3	Downstream	29412
20	AX-76199784	10983967	C	T	1.60E-22	Dup 1	EDN3	Upstream	17587
20	AX-76199800	10991662	C	T	1.60E-22	Dup 1	EDN3	Upstream	9892
20	AX-76199789	10987331	G	A	3.09E-22	Dup 1	EDN3	Upstream	14223
20	AX-76201355	11567733	T	C	3.54E-22	Dup 2	C20H20ORF85	Upstream	55096
20	AX-76199785	10985503	C	T	3.84E-22	Dup 1	EDN3	Upstream	16051
20	AX-76199896	11023595	A	G	8.58E-21	Dup 1	EDN3	Downstream	22041
20	AX-76201424	11592590	T	C	8.58E-21	Dup 2	C20H20ORF85	Upstream	79953
20	AX-76201452	11605373	A	C	8.58E-21	Dup 2	C20H20ORF85	Upstream	92736
20	AX-80927854	11625060	C	G	5.66E-17		C20H20ORF85	Upstream	112423
20	AX-76199911	11029089	T	C	9.90E-17	Dup 1	EDN3	Downstream	27535
20	AX-76199682	10953688	A	G	1.61E-16		SNRPN	Synon	0
20	AX-76201512	11627335	G	A	7.52E-16	Dup 2	C20H20ORF85	Upstream	114698
20	AX-76200161	11113512	A	G	3.00E-15		TH1L	Upstream	780
20	AX-76200189	11125617	C	T	3.00E-15		GNAS	Intron	0
20	AX-80853401	10965771	C	G	3.16E-15		DDX27	Upstream	3835
20	AX-76199675	10950531	C	A	3.23E-15		ELMO2	Upstream	3897
20	AX-80815822	10905641	C	G	3.23E-15		CDH22	Upstream	20000
20	AX-76200001	11058629	C	T	3.95E-15	Dup 1	SLMO2	Upstream	15512
20	AX-76200035	11069818	T	C	4.81E-15	Dup 1	SLMO2	Upstream	4323
20	AX-80868985	11078765	C	G	5.80E-15		SLMO2	UTR-3	0
20	AX-76199666	10947464	A	G	7.33E-15		ELMO2	Upstream	830
20	AX-76200905	11395531	A	C	7.33E-15		VAPB	Intron	0
20	AX-76199599	10922992	G	A	9.86E-15		CDH22	Upstream	37351
20	AX-76199979	11049397	T	C	1.34E-14	Dup 1	ZNF831	Downstream	1763
20	AX-76199981	11049513	T	C	3.72E-14	Dup 1	ZNF831	Downstream	1647

### Detection of Signatures of Selection in Pairwise Populations of DCL and RCL

**Figure 5:**
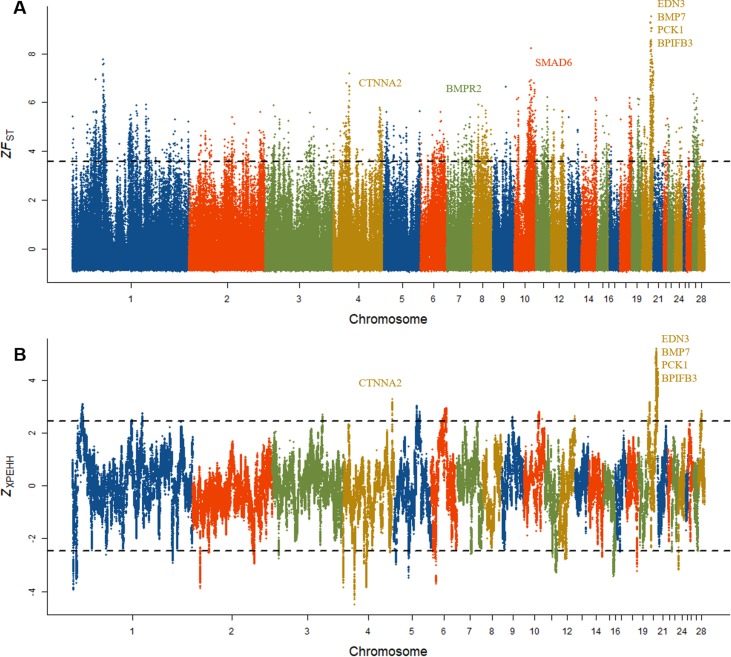
Signature of selection in Dongxiang blue-shelled chicken. **(A)** Genomic landscape of signature of selection as measured by population differentiation (*F*_ST_) in pairwise comparisons of DCL and RCL and the outlier SNPs on chromosome 20 showing extremely high *ZF*_ST_ values harboring *EDN3* and *BMP7* genes. **(B)** Genomic distribution of signatures of selection as measured by cross-population extended haplotype homozygosity (XP-EHH) test.

To determine whether a positive selection might have occurred recently in Dongxiang blue-shelled chicken, two independent methods *F*_ST_ and XP-EHH were implemented to identify the signatures of selection between DCL and RCL. We first estimated the F_ST_ to identify candidate genes that potentially regulate specific divergent traits in DCL and RCL. We standardized the *F*_ST_ to *Z* scores and considered the upper tail (top 1%) of *ZF*_ST_ distribution as the outlier SNPs and, thus, were considered to be potentially positively selected. There were 3,779 outlier SNPs with *ZF*_ST_ of >3.5 between DCL and RCL (**Figure 5A**), and 34 outlier SNPs with extremely high *ZF*_ST_ values (top 0.01%) were identified, which formed a potentially differentiated region on chromosome 20. A total of 813 genes were annotated according to the outliers based on the University of California—Santa Cruz database. Within the region with the highest *ZF*_ST_ value, four genes, viz., *PCK1*, *BPIFB3*, *EDN3*, and *BMP7* with important functions in physiological processes located on chromosome 20 were identified (**Table 3**).

**Table 3:** Summary of the most interesting candidate genes within extreme signatures.

Chr	Position (Mb)	Method	Candidate gene	Gene function or full name
4	87.8–87.9	F_ST_, XP-EHH	CTNNA2	Proliferation of chicken primordial germ cells
7	11.9–12.0	F_ST_	BMPR2	Bone morphogenetic protein receptor type 2
10	18.7–18.8	F_ST_	SMAD6	Chondrocyte differentiation; neuronal differentiation
20	10.5–10.6	F_ST_, XP-EHH	BPIFB3	Avian egg natural defenses
20	10.9–11.1	F_ST_, XP-EHH	EDN3	Promotes neural crest cell proliferation and mediates a vast increase in melanocyte number.
20	11.0–11.1	F_ST_, XP-EHH	SLMO2	PRELI domain containing 3B
20	11.0–11.1	F_ST_, XP-EHH	TUBB1	Tubulin beta 1 class VI
20	11.3–11.4	F_ST_, XP-EHH	VAPB	VAMP associated protein B and C
20	11.7–11.8	F_ST_, XP-EHH	PCK1	Associated with egg production
20	11.8–12.1	F_ST_, XP-EHH	BMP7	Associated with hyperpigmentation of visceral peritoneum; function in follicular development

We also employed the XP-EHH method to explore potential variants experiencing selection between DCL and RCL. Similar to the F_ST_ method, we calculated and standardized XP-EHH data and considered the upper tail of *Z*_XP-EHH_ distribution as mentioned previously. We identified 3,225 outlier SNPs with *Z*_XP-EHH_ of >2.5 (**Figure 5B**) and annotated 149 genes. The XP-EHH analysis also showed the extreme outliers with a high Z_XP-EHH_ located on chromosome 20, comprising the four genes *EDN3*, *BMP7*, *PCK1*, and *BPIFB3*.

As expected, each approach detected regions displaying selection signatures according to the feature of genetic polymorphism data. We compared the overlap between outlier SNPs and genes in pairwise populations of DCL and RCL identified by the two methods. The region on chromosome 20 was mostly distinct from others in the genome. Of the 3,779 outliers detected by F_ST_ sweep mapping, 305 overlapped the outliers that were detected by the XP-EHH method (**Supplementary Figure 3B**), and 253 of the overlapping outliers were detected by both methods are distributed on chromosome 20. Sixty-one genes were found to overlap, as detected by the two methods, which also included the four genes *EDN3*, *BMP7*, *PCK1*, and *BPIFB3* on chromosome 20 (**Supplementary Figure 3C**). The distribution of potentially selected outlier SNPs in the genes was examined. Most of the outliers were located in introns, untranslated regions, and intergenic regions (**Supplementary Figure 3A**), suggesting that the potential target for selection was regulatory mutation.

### Gene Enrichment Analysis

The potential selected regions contained 813 and 149 genes in the top 1% outliers detected by F_ST_ and XP-EHH approaches, respectively. Analysis of gene enrichment with the clusters of genes presented significant categories potentially involved in melanin pigmentation and reproduction (**Table 4**). Twenty significantly enriched groups of multiple categories were found (**Supplementary Figure 4**). The highly significant category was “regulation of protein kinase activity” (GO: 0045859) (*P* = 5.95E-8) containing the candidate genes *PCK1* and *EDN3*; another significant category was “transmembrane receptor protein tyrosine kinase signaling pathway” (GO: 0007169) (*P* = 5.80E-9). Two significant categories “neural crest cell differentiation” (GO: 0014033) (*P* = 5.88E-4) and “neural crest cell migration” (GO: 0001755) (*P* = 1.29E-3) related to BPs of neural crest cell involved the two genes *EDN3* and *BMP7*.

**Table 4:** Gene enrichment analysis showing the functions of important genes in melanogenesis and folliculogenesis.

Term	Type	Description	*P* value	Gene counts	Method
GO:0007169	BP	Transmembrane receptor protein tyrosine kinase signaling pathway	5.80E-09	68	*F*_ST_
GO:0009792	BP	Embryo development ending in birth or egg hatching	5.39E-08	57	*F*_ST_
GO:0045859	BP	Regulation of protein kinase activity	5.95E-08	70	*F*_ST_
GO:0030335	BP	Positive regulation of cell migration	2.96E-07	49	*F*_ST_
hsa04310	KEGG pathway	Wnt signaling pathway	8.32E-06	20	*F*_ST_
GO:0051216	BP	Cartilage development	1.46E-05	24	*F*_ST_
GO:0014032	BP	Neural crest cell development	5.69E-04	5	XP-EHH
GO:0061458	BP	Reproductive system development	6.89E-04	35	*F*_ST_
GO:0014033	BP	Neural crest cell differentiation	8.88E-04	5	XP-EHH
GO:0001755	BP	Neural crest cell migration	1.29E-03	4	XP-EHH
GO:0071383	BP	Cellular response to steroid hormone stimulus	1.92E-03	8	XP-EHH
GO:0045859	BP	Regulation of protein kinase activity	3.18E-03	15	XP-EHH
GO:0032870	BP	Cellular response to hormone stimulus	7.19E-03	13	XP-EHH

We also found two significant categories related to skeletal system development, namely, “bone development” (GO: 0060348) (*P* = 2.32E-7) and “cartilage development” (GO: 0051216) (*P* = 1.46E-7), involving the genes *BMP7* and bone morphogenetic protein receptor type 2 (*BMPR2*) (**Table 4**). Among the candidate genes, we found that *BMP7* and *BMPR2* are also involved in the GO BPs category “reproductive system development” (GO: 0061458) (*P* = 6.89 E-4); *BMP7* and *PCK1* are involved in the “cellular response to steroid hormone stimulus” (GO: 0071383) (*P* = 1.92E-3) and “cellular response to hormone stimulus” (GO: 0032870) (*P* = 7.19E-3). The categories identified were closely related to reproduction.

### Association Analysis Between Selected Loci and Egg Production

To test the potentially pleiotropic effects on egg production, we selected a cluster of SNPs underlying selection signatures identified by the independent selective sweep analysis to perform association analysis for egg production trait. Thirty-seven SNPs were identified to be significantly associated with the number of eggs. These SNPs are distributed closely on chromosome 20, which covered a 2.4-Mb region from 10,785,456 to 13,243,956 bp, including the *EDN3* and *BMP7* genes (**Supplementary Table 3**). This suggests that the genomic region has a strong effect on egg production.

### Validation of Genomic Duplication in Associated Candidate Region

To verify whether dermal hyperpigmentation in the comb of Dongxiang blue-shelled chicken is caused by the inversion of genomic duplication, a multiple PCR diagnostic test was performed to detect the boundaries of the two duplications. We identified two boundaries of the two duplications with an inversion of the second duplication in DCL successfully, whereas no duplication was found in RCL (**Supplementary Figure 5**). We confirmed that the dark comb color in Dongxiang blue-shelled chicken is caused by the genomic rearrangement on chromosome 20.

### Expression of Candidate Genes in the Ovary

**Figure 6:**
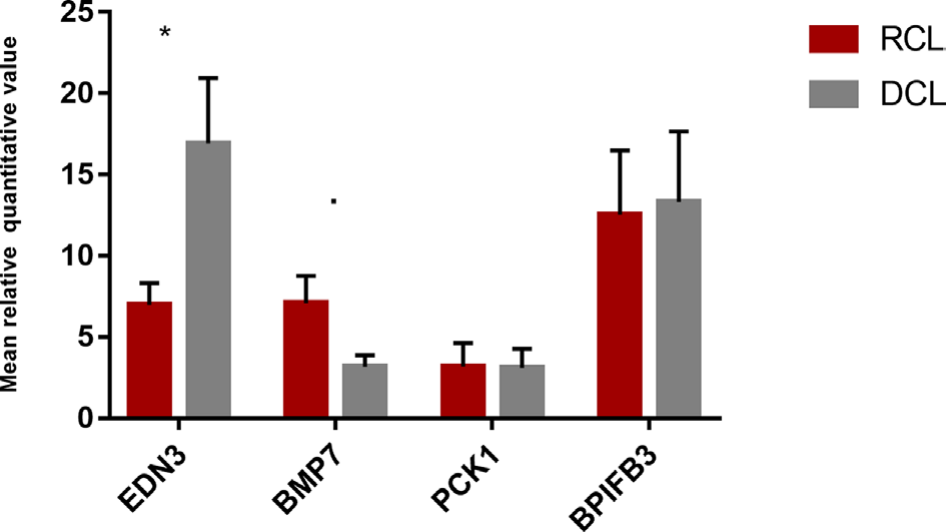
Expression analysis of candidate genes. The quantitative real-time polymerase chain reaction (qPCR) analysis of four genes (*EDN3*, *BMP7*, *PCK1*, and *BPIFB3*) in the ovary tissue of dark comb and red comb chickens. The error bars indicate the standard error of the mean. *p < 0.05,·*p* < 0.1.

To access the potential effect of the four candidate genes (*EDN3*, *BMP7*, *PCK1*, and *BPIFB3*) on egg production, the qPCR was performed using cDNA obtained from the ovary of dark comb and red comb layers. We found that *EDN3* was overexpressed in the ovary of dark comb layers (*P* = 0.041, ∼2.5-fold increase), suggesting that the genomic duplication close to the *EDN3* gene act as an enhancer in regulating the function, whereas an approximate twofold decrease was observed in the expression of *BMP7* in the ovary of dark comb layers, but the difference is marginally nonsignificant (*P* = 0.061). No significant differential expression was observed with respect to *PCK1* and *BPIFB3* in the ovary between DCL and RCL (**Figure 6**).

## Discussion

Long-term divergent selection has genome-wide effects on phenotypic variation, such as the body weight (Johansson et al., 2010; Lillie et al., 2018) and abdominal fat content (Zhang et al., 2012) in chickens. The chickens used in the present study have been separated for over 20 generations and subjected to divergent selection for comb color, resulting in a significant difference in the number of eggs and color of the comb, which can be an indicator of egg production. In the present study, through genomic analyses of three lines of Dongxiang blue-shelled chicken, we elucidated the extent of genomic changes in the three lines resulting from selection and identified a genomic region associated with dermal hyperpigmentation in chicken, which is also involved in ovarian function. We demonstrated the pleiotropic alleles at *EDN3* and *BMP7* potentially explain the link between comb color and egg production.

Dark appearance is widespread among livestock due to melanin migration and deposition in the dermal layer of tissues. Two loci are related to dermal hyperpigmentation, with the autosomal dominant nature of FM allele and the sex-linked inhibitor of dermal melanin (ID) locus on the Z chromosome (Dorshorst et al., 2011). A previous GWAS demonstrated that a locus of the *EDN3* gene on chromosome 20 and a region on chromosome 21 are significantly associated with the comb color trait in two Swedish local chicken breeds (Johansson and Nelson, 2015). Furthermore, six SNPs on chromosome Z has been reported to segregate with the comb color phenotype in one of the breeds (Johansson and Nelson, 2015), which is inconsistent with the genomic region of chromosome Z associated with the ID locus in other breeds (Dorshorst et al., 2010). In the present study, we found that dermal hyperpigmentation in chicken comb is associated with the *EDN3* gene, which is involved in genomic duplication. We also identified the *BMP7* gene located on chromosome 20 with a potential role in melanin pigmentation. Previous studies have shown that *EDN3* gene was associated with dermal hyperpigmentation (Dorshorst et al., 2011; Shinomiya et al., 2012), and the *BMP7* gene was associated with hyperpigmentation of the visceral peritoneum (Luo et al., 2013). No other significant regions were found to be associated with comb color phenotype in the current study. This indicates that the locus of ID is homozygous in blue eggshell chickens. The different loci contributing to dark comb probably present a case of parallel selection at the molecular level under adaptive selection. As a matter of fact, the common phenotypes in different breeds are probably caused by various loci, such as frizzle feather (Guo et al., 2018), blue eggshell (Wragg et al., 2013), polydactyly (Zhang et al., 2016), and dwarfism (Wang et al., 2017) in different chicken breeds. Thus, considering the genetic architectures of different chicken populations is indispensable even though they have the same phenotypic trait.

As an important secondary sexual characteristic, the comb has a close relationship with the production and reproduction traits in chickens (Cornwallis and Birkhead, 2007; Mukhtar and Khan, 2012). In this study, we have shown that comb color was a significant predictor of egg production in Dongxiang blue-shelled chicken. The female chickens with red comb had higher number of eggs. We scanned genome-wide selective signatures in DCL and RCL and identified two genes, viz., *EDN3* and *BMP7*, with pleiotropic effects on melanin pigmentation and reproduction, which potentially cause the link between comb color and egg production. Gene enrichment analysis showed significant categories involved in dermal hyperpigmentation and reproduction. Neural crest cell system and regulation of protein kinase activity are involved in the significant GO categories, which are associated with the process of melanocytes migration (Sommer, 2011; Shinomiya et al., 2012) and melanin synthesis (Hearing, 2011). GO categories that closely relate to bone development and cellular response to hormone stimulus are also involved. Bone metabolism has been shown to be linked with egg production, for instance, female chickens deposit calcium in the diaphysis, where it can be transferred into the eggshell (Johnsson et al., 2012; Wright et al., 2012). *EDN3* and *BMP7* genes are enriched in the categories, suggesting their important roles in melanin pigmentation and reproduction.

Endothelins (EDN) are 21-amino acid vasoactive peptides reported to play various roles in the reproduction system such as in steroidogenesis, folliculogenesis, and ovulation (Ervin et al., 2017). The* EDN3* gene has been reported to influence the function of granulosa cells in several mammals. This can significantly suppress estrogen production induced by follicular-stimulating hormone (FSH) (Calogero et al., 1998; Ervin et al., 2017). The expression of *EDN3* can be regulated by gonadotropins in epithelial cells of the oviduct, particularly increased in the isthmus of this tissue (Jeoung et al., 2010). Moreover, *EDN3* has been reported to play important roles in melanogenesis; it is required for the development of neural crest-derived pigment cells (like melanocytes) (Baynash et al., 1994), promoting neural crest cell proliferation and consequently increasing the number of melanocytes significantly (Lahav et al., 1996). In the present study, we found that the expression of *EDN3* is increased in the ovary of dark comb layers (**Figure 6**). Based on the pleiotropic effects of *EDN3*, we inferred that the upregulated expression of *EDN3* increased melanogenesis and inhibited folliculogenesis, resulting in the association between comb color and egg production. The *BMP7* gene has known functions in folliculogenesis and ovulation in mammals (Lee et al., 2001; Shimasaki et al., 2004; Rossi et al., 2016) and chickens (Onagbesan et al., 2003). The *BMP7* gene could increase the expression of follicle-stimulating hormone receptor (*FSHR*) gene in human granulosa cells and decrease the expression of luteinizing hormone receptor (*LHR*) gene (Shi et al., 2010). It also increases estradiol production by stimulating the activity of FSH (Shimasaki et al., 1999). In chickens, *BMP7* can help follicular development by stimulating granulosa cell proliferation (Onagbesan et al., 2003). On the contrary, decreased *BMP7* expression can disrupt melanocyte homeostasis in normal melanocytes and inhibit tumor growth in human uveal melanomas (Notting et al., 2007), whereas upregulation of *BMP7* correlates with tumor progression (Hsu et al., 2005). In the present study, we found downregulation of *BMP7* expression in the ovary of dark comb chickens, which might potentially be associated with egg production and melanin pigmentation.

During the past 10 years, GWAS have identified significant associations with many complex traits in humans and livestock; several variants affect multiple traits (Paaby and Rockman, 2013; Pickrell et al., 2016). Pleiotropy is a well-studied phenomenon with respect to a single mutation affecting multiple phenotypes. For instance, the comb mass has been shown to be linked with egg production and bone allocation in chicken. A locus containing two tightly linked genes produces pleiotropic effects on such related phenotypes (Johnsson et al., 2012). Rose-comb with homozygous loci in males has been associated with poor sperm motility. It has been reported that rose-comb is caused by a large genomic inversion, which induces ectopic expression of the *MNR2* gene. Simultaneously, the genomic structure variation disrupts the *CCDC108* gene located at the distal inversion breakpoint, affecting sperm motility (Imsland et al., 2012). Gray hair is associated with a high incidence of melanoma in horses. It has also been found that gray hair phenotype is caused by a 4.6-kb duplication in the *STX17* gene constituting a cis-acting regulatory mutation, causing susceptibility to melanoma (Rosengren Pielberg et al., 2008). Several examples of structure variation contributing to single gene traits have previously been described in chicken (Wright et al., 2009; Wang et al., 2013; Dorshorst et al., 2015; Guo et al., 2016). This suggests that structural variation in the genome has range effects on the evolution of phenotypic diversity. Indeed, the structural genomic variations play important roles in adaptation and diversification both in animals and plants (Wellenreuther et al., 2019), for instance, a large structural variation (specifically an inversion) that harbors genes controlling color-pattern leads to an accentuated differentiation between ecotypes in stick insect (Lucek et al., 2019). As the structural variation along the genome with long fragment frequently spans more than one gene, it is plausible that the changes in genome can cause wide effects on multiple genes. We identified the genomic region encompassing two duplications, which has been reported to be the causal mutation of dermal hyperpigmentation (Dorshorst et al., 2011). We also identified two genes, namely* EDN3* and *BMP7*, which are in or close to the region of duplication. We speculate that the structural variation probably influences the expression of genes harbored in the genomic region, although the effects of such genes must be validated through functional approaches.

In most avian species, the males with the most impressive sexual ornaments are favored by females, thereby siring more offspring than their competitors (Tobias et al., 2012). For instance, males with larger comb size were preferred by females because larger combs are a cue for females, indicating the sire quality of the male (Mukhtar and Khan, 2012). The relationship between comb color and egg production potentially provides a case of sexual selection. If females were choosing males with redder comb, then this would support phenotype-linked fertility hypothesis, which suggests a link between a male secondary sexual trait and the functional ability of its sperm (Sheldon, 1994), because comb color was positively related to sperm viability (Navara et al., 2012). Interestingly, the genetic basis of such sexual traits has been explicated in part by the mechanism of pleiotropy (Chenoweth and McGuigan, 2010). Although in the case of domesticated chicken, which is no longer subject to sexual selection, the preexisting genetic architecture of the ancestral red jungle fowl will be maintained by the current artificial selection (Johnsson et al., 2012) and could orchestrate trade-offs of sexual signals.

## Conclusions

In conclusion, the results showed that there is a close relationship between comb color and egg production. The two loci on chromosome 20 (*EDN3* and *BMP7*) potentially contributed to comb color phenotype. Further analyses of genomic data suggested that the GO terms related to melanogenesis and folliculogenesis are important targets of selection for hyperpigmentation and egg production in Dongxiang blue-shelled chickens. Both *EDN3* and *BMP7* genes play pleiotropic roles in hyperpigmentation and egg production in chicken. Our study provides novel insights on pleiotropy of chicken comb-related genes and necessitates further selection and breeding in egg production.

## Data Availability Statement

All the raw data of SNP genotyping for the study population were submitted to the National Center for Biotechnology Information Gene Expression Omnibus database with the Gene Expression Omnibus accession no. GSE124906.

## Ethics Statement

Regular quarantine inspection of the experimental farm in China Agricultural University was conducted. The blood samples were collected from brachial veins of chickens by standard venipuncture. The whole procedure was carried out strictly following the protocol approved by the Animal Care Committee of China Agricultural University.

## Author Contributions

XD: data analysis, experiments performing, manuscript writing and editing; JL: study design assessment, animal resources supporting; YZ: data analysis; DH: experiments performing; GH: samples recruitment; JW: samples recruitment; XD: study design assessment, manuscript writing; CW: study design assessment. All authors have reviewed and approved the manuscript.

## Funding

This work was supported in part by grants from National Scientific Supporting Projects of China (2013AA102501), National Nature Science Foundation (31872316 and 31472082), and the China Agriculture Research System (CARS-41).

## Conflict of Interest Statement

The authors declare that the research was conducted in the absence of any commercial or financial relationships that could be construed as a potential conflict of interest.

## Supplementary Material

Supplementary Figure 1 Comparison of blood parameters between red comb and dark comb chickens. No significant difference was observed in the three parameters hemoglobin, heme, and iron (Fe) ion.

Supplementary Figure 2 Tissue distribution of melanin in Dongxiang blue-shelled chicken. Comb tissues from RCL (A, C) and DCL (B, D) (A and B with scale bar = 200 μm, C and D with scale bar = 50 μm.). Melanin was distributed in the tissue of DCL whereas it was absent in RCL. Hematoxylin and eosin staining.

Supplementary Figure 3 Summary of outlier SNPs and genes from the identification of signature of selection. (A) Distribution of outliers in the gene structure detected by the FST and XP-EHH tests. Overlaps of outlier SNPs (B) and genes (C) detected by the FST and XP-EHH tests.

Supplementary Figure 4 Gene enrichment analysis. (A) The category clusters from XP-EHH test. (B) The category clusters from FST test.

Supplementary Figure 5 Diagnostic test of boundaries of both the duplicated regions. The dark comb chickens displayed two bands in the agarose gel indicating the duplications in the genome, whereas the red comb chickens without the variant of genomic structure showed one band in the gel.

## References

[B1] Andersson L. Georges M. (2004). Domestic-animal genomics: deciphering the genetics of complex traits. Nat. Rev. Genet. 5 (3), 202–212. 10.1038/nrg1294 14970822

[B2] Baynash A. G. Hosoda K. Giaid A. Richardson J. A. Emoto N. Hammer R. E. (1994). Interaction of endothelin-3 with endothelin-B receptor is essential for development of epidermal melanocytes and enteric neurons. Cell 79 (7), 1277–1285. 10.1016/0092-8674(94)90018-3 8001160

[B3] Calogero A. E. Burrello N. Ossino A. M. (1998). Endothelin (ET)-1 and ET-3 inhibit estrogen and cAMP production by rat granulosa cells in vitro. J. Endocrinol. 157 (2), 209–215. 10.1677/joe.0.1570209 9659283

[B4] Chenoweth S. F. McGuigan K. (2010). The genetic basis of sexually selected variation. Ann. Rev. Ecol. Evol. Syst. 41 (1), 81–101. 10.1146/annurev-ecolsys-102209-144657

[B5] Cornwallis C. K. Birkhead T. R. (2007). Experimental evidence that female ornamentation increases the acquisition of sperm and signals fecundity. Proc. Biol. Sci. 274 (1609), 583–590. 10.1098/rspb.2006.3757 17476780 PMC1766391

[B6] Crawford R. D. Smyth J. R. Jr. (1964). Semen quality and the gene for rose comb in the domestic fowl1. Poultr. Sci. 43 (6), 1551–1557. 10.3382/ps.0431551 14208710

[B7] Curik I. Druml T. Seltenhammer M. Sundstrom E. Pielberg G. R. Andersson L. (2013). Complex inheritance of melanoma and pigmentation of coat and skin in Grey horses. PLoS Genet. 9 (2), e1003248. 10.1371/journal.pgen.1003248 23408897 PMC3567150

[B8] Danecek P. Auton A. Abecasis G. Albers C. A. Banks E. DePristo M. A. (2011). The variant call format and VCFtools. Bioinformatics 27 (15), 2156–2158. 10.1093/bioinformatics/btr330 21653522 PMC3137218

[B9] Dorshorst B. Harun-Or-Rashid M. Bagherpoor A. J. Rubin C. J. Ashwell C. Gourichon D. (2015). A genomic duplication is associated with ectopic eomesodermin expression in the embryonic chicken comb and two duplex-comb phenotypes. PLoS Genet. 11 (3), e1004947. 10.1371/journal.pgen.1004947 25789773 PMC4366209

[B10] Dorshorst B. Molin A. M. Rubin C. J. Johansson A. M. Stromstedt L. Pham M. H. (2011). A complex genomic rearrangement involving the endothelin 3 locus causes dermal hyperpigmentation in the chicken. PLoS Genet. 7 (12), e1002412. 10.1371/journal.pgen.1002412 22216010 PMC3245302

[B11] Dorshorst B. Okimoto R. Ashwell C. (2010). Genomic regions associated with dermal hyperpigmentation, polydactyly and other morphological traits in the Silkie chicken. J. Hered. 101 (3), 339–350. 10.1093/jhered/esp120 20064842

[B12] Ervin J. M. Schutz L. F. Spicer L. J. (2017). Current status of the role of endothelins in regulating ovarian follicular function: a review. Anim. Reprod. Sci. 186, 1–10. 10.1016/j.anireprosci.2017.09.008 28967452

[B13] Guo X. Li Y. Q. Wang M. S. Wang Z. B. Zhang Q. Shao Y. (2018). A parallel mechanism underlying frizzle in domestic chickens. J. Mol. Cell. Biol. 10 (6), 589–591. 10.1093/jmcb/mjy037 29868726

[B14] Guo Y. Gu X. Sheng Z. Wang Y. Luo C. Liu R. (2016). A complex structural variation on chromosome 27 leads to the ectopic expression of HOXB8 and the muffs and beard phenotype in chickens. PLoS Genet. 12 (6), e1006071. 10.1371/journal.pgen.1006071 27253709 PMC4890787

[B15] Han D. Wang S. Hu Y. Zhang Y. Dong X. Yang Z. (2015). Hyperpigmentation results in aberrant immune development in silky fowl (Gallus gallus domesticus Brisson). PLoS One 10 (6), e0125686. 10.1371/journal.pone.0125686 26047316 PMC4457905

[B16] Hearing V. J. (2011). Determination of melanin synthetic pathways. J. Invest. Dermatol. 131 (E1), E8–e11. 10.1038/skinbio.2011.4 22094404 PMC6944209

[B17] Hodgkin J. (1998). Seven types of pleiotropy. Int. J. Dev. Biol. 42 (3), 501–505. 9654038

[B18] Hongtao L. (2006). Analysis of genetic diversity and study of conservation and breeding strategies in Dehua Black chicken (in Chinese). Chin. Livestock Poultr Breed. 12, 2.

[B19] Hsu M. Y. Rovinsky S. Penmatcha S. Herlyn M. Muirhead D. (2005). Bone morphogenetic proteins in melanoma: angel or devil? Cancer Metastasis Rev. 24 (2), 251–263. 10.1007/s10555-005-1575-y 15986135

[B20] Imsland F. Feng C. Boije H. Bed’hom B. Fillon V. Dorshorst B. (2012). The Rose-comb mutation in chickens constitutes a structural rearrangement causing both altered comb morphology and defective sperm motility. PLoS Genet. 8 (6), e1002775. 10.1371/journal.pgen.1002775 22761584 PMC3386170

[B21] Jeoung M. Lee S. Hawng H. K. Cheon Y. P. Jeong Y. K. Gye M. C. (2010). Identification of a novel role for endothelins within the oviduct. Endocrinology 151 (6), 2858–2867. 10.1210/en.2009-1155 20357223 PMC2875811

[B22] Johansson A. M. Nelson R. M. (2015). Characterization of genetic diversity and gene mapping in two Swedish local chicken breeds. Front. Genet. 6, 44. 10.3389/fgene.2015.00044 25741364 PMC4330917

[B23] Johansson A. M. Pettersson M. E. Siegel P. B. Carlborg O. (2010). Genome-wide effects of long-term divergent selection. PLoS Genet. 6 (11), e1001188. 10.1371/journal.pgen.1001188 21079680 PMC2973821

[B24] Johnsson M. Gustafson I. Rubin C. J. Sahlqvist A. S. Jonsson K. B. Kerje S. (2012). A sexual ornament in chickens is affected by pleiotropic alleles at HAO1 and BMP2, selected during domestication. PLoS Genet. 8 (8), e1002914. 10.1371/journal.pgen.1002914 22956912 PMC3431302

[B25] Johnsson M. Rubin C. J. Hoglund A. Sahlqvist A. S. Jonsson K. B. Kerje S. (2014). The role of pleiotropy and linkage in genes affecting a sexual ornament and bone allocation in the chicken. Mol. Ecol. 23 (9), 2275–2286. 10.1111/mec.12723 24655072

[B26] Kinsella R. J. Kahari A. Haider S. Zamora J. Proctor G. Spudich G. (2011). Ensembl BioMarts: a hub for data retrieval across taxonomic space. Database (Oxford) 2011, bar030. 10.1093/database/bar030 21785142 PMC3170168

[B27] Kranis A. Gheyas A. A. Boschiero C. Turner F. Yu L. Smith S. (2013). Development of a high density 600K SNP genotyping array for chicken. BMC Genomics 14, 59. 10.1186/1471-2164-14-59 23356797 PMC3598943

[B28] Lahav R. Ziller C. Dupin E. Le Douarin N. M. (1996). Endothelin 3 promotes neural crest cell proliferation and mediates a vast increase in melanocyte number in culture. Proc. Natl. Acad. Sci. U.S.A. 93 (9), 3892–3897. 10.1073/pnas.93.9.3892 8632985 PMC39455

[B29] Lee W. S. Otsuka F. Moore R. K. Shimasaki S. (2001). Effect of bone morphogenetic protein-7 on folliculogenesis and ovulation in the rat. Biol. Reprod. 65 (4), 994–999. 10.1095/biolreprod65.4.994 11566718

[B30] Lillie M. Sheng Z. Y. Honaker C. F. Andersson L. Siegel P. B. Carlborg O. (2018). Genomic signatures of 60 years of bidirectional selection for 8-week body weight in chickens. Poultr. Sci. 97 (3), 781–790. 10.3382/ps/pex383 29272516

[B31] Lucek K. Gompert Z. Nosil P. (2019). The role of structural genomic variants in population differentiation and ecotype formation in Timema cristinae walking sticks. Mol. Ecol. 28 (6), 1224–1237. 10.1111/mec.15016 30636326

[B32] Luo C. Qu H. Wang J. Wang Y. Ma J. Li C. (2013). Genetic parameters and genome-wide association study of hyperpigmentation of the visceral peritoneum in chickens. BMC Genomics 14, 334. 10.1186/1471-2164-14-334 23679099 PMC3663821

[B33] Mukhtar N. Khan S. H. (2012). Comb: an important reliable visual ornamental trait for selection in chickens. Worlds Poultr. Sci. J. 68 (3), 425–434. 10.1017/S0043933912000542

[B34] Navara K. J. Anderson E. M. Edwards M. L. (2012). Comb size and color relate to sperm quality: a test of the phenotype-linked fertility hypothesis. Behav. Ecol. 23 (5), 1036–1041. 10.1093/beheco/ars068

[B35] Notting I. Buijs J. Mintardjo R. van der Horst G. Vukicevic S. Lowik C. (2007). Bone morphogenetic protein 7 inhibits tumor growth of human uveal melanoma in vivo. Invest. Ophthalmol. Vis. Sci. 48 (11), 4882–4889. 10.1167/iovs.07-0505 17962434

[B36] Onagbesan O. M. Bruggeman V. Van As P. Tona K. Williams J. Decuypere E. (2003). BMPs and BMPRs in chicken ovary and effects of BMP-4 and -7 on granulosa cell proliferation and progesterone production in vitro. Am. J. Physiol. Endocrinol. Metab. 285 (5), E973–983. 10.1152/ajpendo.00104.2003 12888485

[B37] Paaby A. B. Rockman M. V. (2013). The many faces of pleiotropy. Trends Genet. 29 (2), 66–73. 10.1016/j.tig.2012.10.010 23140989 PMC3558540

[B38] Pickrell J. K. Berisa T. Liu J. Z. Segurel L. Tung J. Y. Hinds D. A. (2016). Detection and interpretation of shared genetic influences on 42 human traits. Nat. Genet. 48 (7), 709–717. 10.1038/ng.3570 27182965 PMC5207801

[B39] Purcell S. Neale B. Todd-Brown K. Thomas L. Ferreira M. A. Bender D. (2007). PLINK: a tool set for whole-genome association and population-based linkage analyses. Am. J. Hum. Genet. 81 (3), 559–575. 10.1086/519795 17701901 PMC1950838

[B40] Rosengren Pielberg G. Golovko A. Sundstrom E. Curik I. Lennartsson J. Seltenhammer M. H. (2008). A cis-acting regulatory mutation causes premature hair graying and susceptibility to melanoma in the horse. Nat. Genet. 40 (8), 1004–1009. 10.1038/ng.185 18641652

[B41] Rossi R. O. Costa J. J. Silva A. W. Saraiva M. V. Van den Hurk R. Silva J. R. (2016). The bone morphogenetic protein system and the regulation of ovarian follicle development in mammals. Zygote 24 (1), 1–17. 10.1017/S096719941400077X 25613521

[B42] Sabeti P. C. Varilly P. Fry B. Lohmueller J. Hostetter E. Cotsapas C. (2007). Genome-wide detection and characterization of positive selection in human populations. Nature 449 (7164), 913–918. 10.1038/nature06250 17943131 PMC2687721

[B43] Scheet P. Stephens M. (2006). A fast and flexible statistical model for large-scale population genotype data: applications to inferring missing genotypes and haplotypic phase. Am. J. Hum. Genet. 78 (4), 629–644. 10.1086/502802 16532393 PMC1424677

[B44] Sheldon B. C. (1994). Male phenotype, fertility, and the pursuit of extra-pair copulations by female birds. Proc. R. Soc. London. Ser B Biol. Sci. 257 (1348), 25–30. 10.1098/rspb.1994.0089

[B45] Shi J. Yoshino O. Osuga Y. Nishii O. Yano T. Taketani Y. (2010). Bone morphogenetic protein 7 (BMP-7) increases the expression of follicle-stimulating hormone (FSH) receptor in human granulosa cells. Fertil. Steril. 93 (4), 1273–1279. 10.1016/j.fertnstert.2008.11.014 19108831

[B46] Shimasaki S. Moore R. K. Otsuka F. Erickson G. F. (2004). The bone morphogenetic protein system in mammalian reproduction. Endocr. Rev. 25 (1), 72–101. 10.1210/er.2003-0007 14769828

[B47] Shimasaki S. Zachow R. J. Li D. Kim H. Iemura S. Ueno N. (1999). A functional bone morphogenetic protein system in the ovary. Proc. Natl. Acad. Sci. U.S.A. 96 (13), 7282–7287. 10.1073/pnas.96.13.7282 10377406 PMC22077

[B48] Shinomiya A. Kayashima Y. Kinoshita K. Mizutani M. Namikawa T. Matsuda Y. (2012). Gene duplication of endothelin 3 is closely correlated with the hyperpigmentation of the internal organs (Fibromelanosis) in silky chickens. Genetics 190 (2), 627–638. 10.1534/genetics.111.136705 22135351 PMC3276631

[B49] Solovieff N. Cotsapas C. Lee P. H. Purcell S. M. Smoller J. W. (2013). Pleiotropy in complex traits: challenges and strategies. Nat. Rev. Genet. 14 (7), 483–495. 10.1038/nrg3461 23752797 PMC4104202

[B50] Sommer L. (2011). Generation of melanocytes from neural crest cells. Pigm. Cell Melanoma Res. 24 (3), 411–421. 10.1111/j.1755-148X.2011.00834.x 21310010

[B51] Stearns F. W. (2010). One hundred years of pleiotropy: a retrospective. Genetics 186 (3), 767–773. 10.1534/genetics.110.122549 21062962 PMC2975297

[B52] Stettenheim P. R. (2000). The integumentary morphology of modern birds: an overview. Am. Zool. 40 (4), 461–477. 10.1093/icb/40.4.461

[B53] Szpiech Z. A. Hernandez R. D. (2014). Selscan: an efficient multithreaded program to perform EHH-based scans for positive selection. Mol. Biol. Evol. 31 (10), 2824–2827. 10.1093/molbev/msu211 25015648 PMC4166924

[B54] Tobias J. A. Montgomerie R. Lyon B. E. (2012). The evolution of female ornaments and weaponry: social selection, sexual selection and ecological competition. Philos. Trans. R. Soc. Lond. B Biol. Sci. 367 (1600), 2274–2293. 10.1098/rstb.2011.0280 22777016 PMC3391421

[B55] Wang M. S. Otecko N. O. Wang S. Wu D. D. Yang M. M. Xu Y. L. (2017). An evolutionary genomic perspective on the breeding of dwarf chickens. Mol. Biol. Evol. 34 (12), 3081–3088. 10.1093/molbev/msx227 28961939

[B56] Wang Z. Qu L. Yao J. Yang X. Li G. Zhang Y. (2013). An EAV-HP insertion in 5’ Flanking region of SLCO1B3 causes blue eggshell in the chicken. PLoS Genet. 9 (1), e1003183. 10.1371/journal.pgen.1003183 23359636 PMC3554524

[B57] Weir B. S. Cockerham C. C. (1984). Estimating f-statistics for the analysis of population structure. Evolution 38 (6), 1358–1370. 10.1111/j.1558-5646.1984.tb05657.x 28563791

[B58] Wellenreuther M. Merot C. Berdan E. Bernatchez L. (2019). Going beyond SNPs: the role of structural genomic variants in adaptive evolution and species diversification. Mol. Ecol. 28 (6), 1203–1209. 10.1111/mec.15066 30834648

[B59] Wragg D. Mwacharo J. M. Alcalde J. A. Wang C. Han J. L. Gongora J. (2013). Endogenous retrovirus EAV-HP linked to blue egg phenotype in Mapuche fowl. PLoS One 8 (8), e71393. 10.1371/journal.pone.0071393 23990950 PMC3747184

[B60] Wright D. Boije H. Meadows J. R. Bed’hom B. Gourichon D. Vieaud A. (2009). Copy number variation in intron 1 of SOX5 causes the Pea-comb phenotype in chickens. PLoS Genet. 5 (6), e1000512. 10.1371/journal.pgen.1000512 19521496 PMC2685452

[B61] Wright D. Rubin C. Schutz K. Kerje S. Kindmark A. Brandstrom H. (2012). Onset of sexual maturity in female chickens is genetically linked to loci associated with fecundity and a sexual ornament. Reprod. Domest Anim. 47 Suppl 1, 31–36. 10.1111/j.1439-0531.2011.01963.x 22212210

[B62] Wright D. Rubin C. J. Martinez Barrio A. Schutz K. Kerje S. Brandstrom H. (2010). The genetic architecture of domestication in the chicken: effects of pleiotropy and linkage. Mol. Ecol. 19 (23), 5140–5156. 10.1111/j.1365-294X.2010.04882.x 21040053

[B63] Yang J. Lee S. H. Goddard M. E. Visscher P. M. (2011). GCTA: a tool for genome-wide complex trait analysis. Am. J. Hum. Genet. 88 (1), 76–82. 10.1016/j.ajhg.2010.11.011 21167468 PMC3014363

[B64] Zhang H. Wang S. Z. Wang Z. P. Da Y. Wang N. Hu X. X. (2012). A genome-wide scan of selective sweeps in two broiler chicken lines divergently selected for abdominal fat content. BMC Genomics 13, 704. 10.1186/1471-2164-13-704 23241142 PMC3557156

[B65] Zhang Z. Nie C. Jia Y. Jiang R. Xia H. Lv X. (2016). Parallel evolution of polydactyly traits in chinese and european chickens. PLoS One 11 (2), e0149010. 10.1371/journal.pone.0149010 26859147 PMC4747547

[B66] Zhou X. Stephens M. (2012). Genome-wide efficient mixed-model analysis for association studies. Nat. Genet. 44 (7), 821–824. 10.1038/ng.2310 22706312 PMC3386377

