# Supplementary figures and images for: Genomic Analysis Reveals Pleiotropic Alleles at EDN3 and BMP7 Involved in Chicken Comb Color and Egg Production
Source: Front Genet. 2019 Jun 28;10:612. doi: 10.3389/fgene.2019.00612 (PMC6611142; doi:10.3389/fgene.2019.00612)

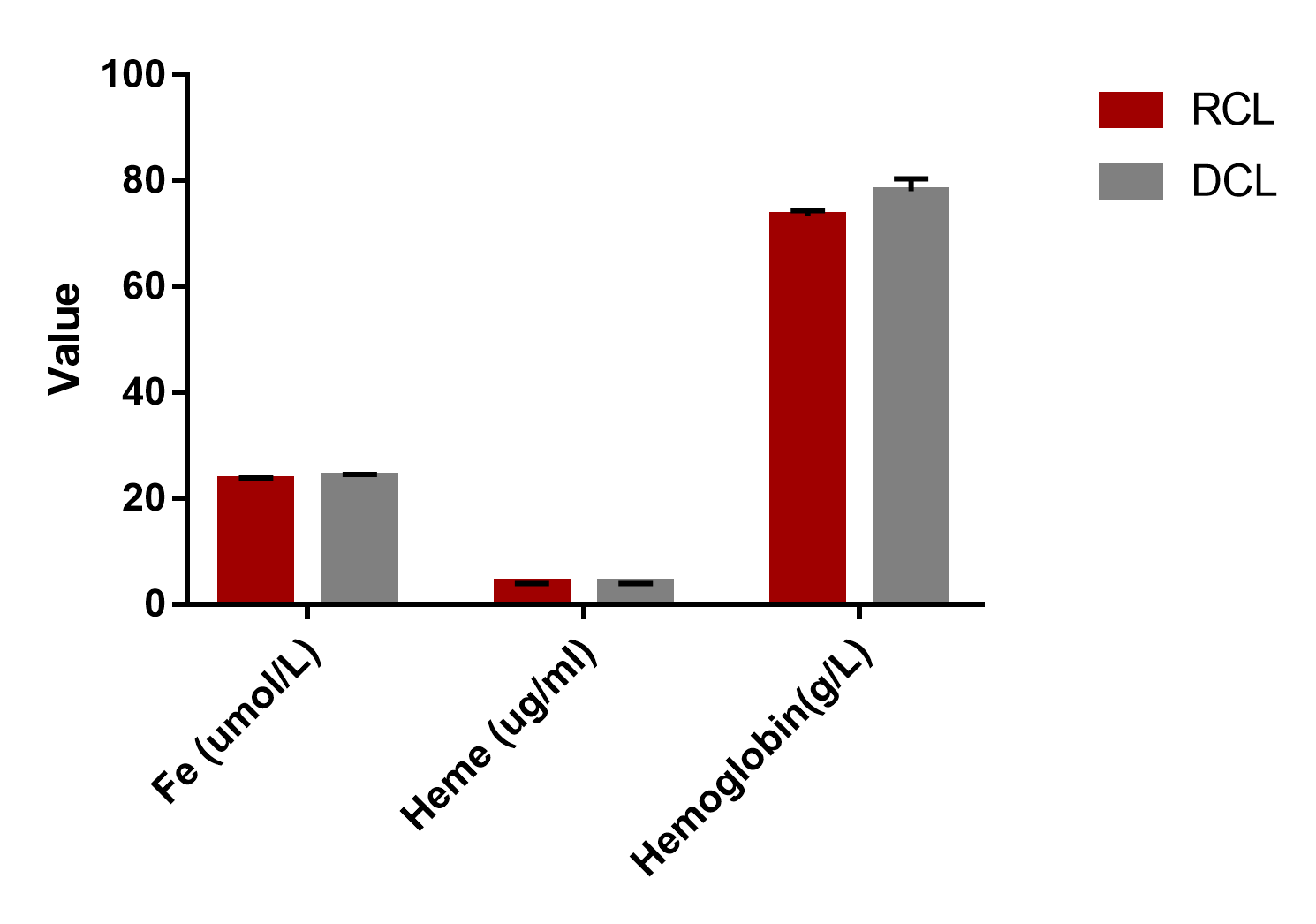

Supplement: Supplementary Figure 1 — Comparison of blood parameters between red comb and dark comb chickens. No significant difference was observed in the three parameters hemoglobin, heme, and iron (Fe) ion. [file Image_1.tif]

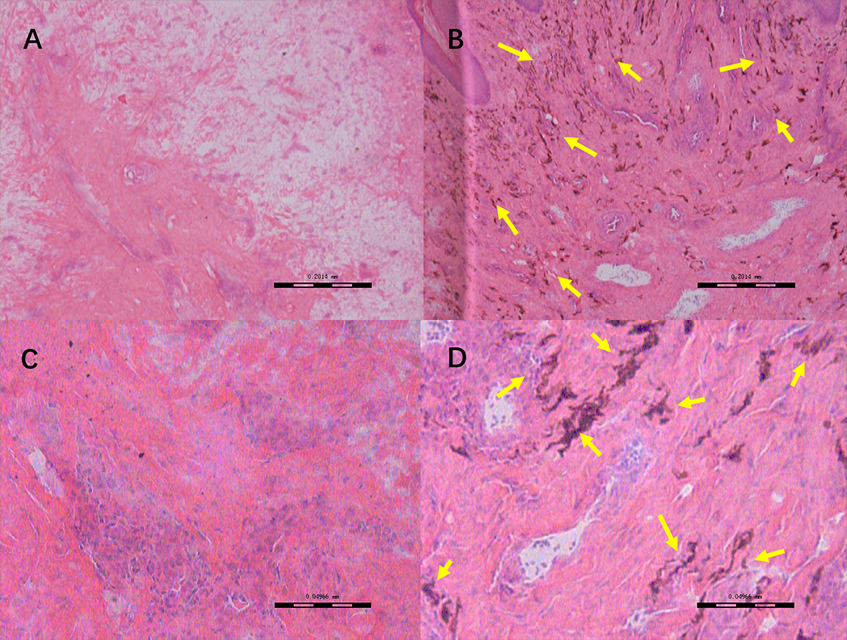

Supplement: Supplementary Figure 2 — Tissue distribution of melanin in Dongxiang blue-shelled chicken. Comb tissues from RCL (A, C) and DCL (B, D) (A and B with scale bar = 200 μm, C and D with scale bar = 50 μm.). Melanin was distributed in the tissue of DCL whereas it was absent in RCL. Hematoxylin and eosin staining. [file Image_2.tif]

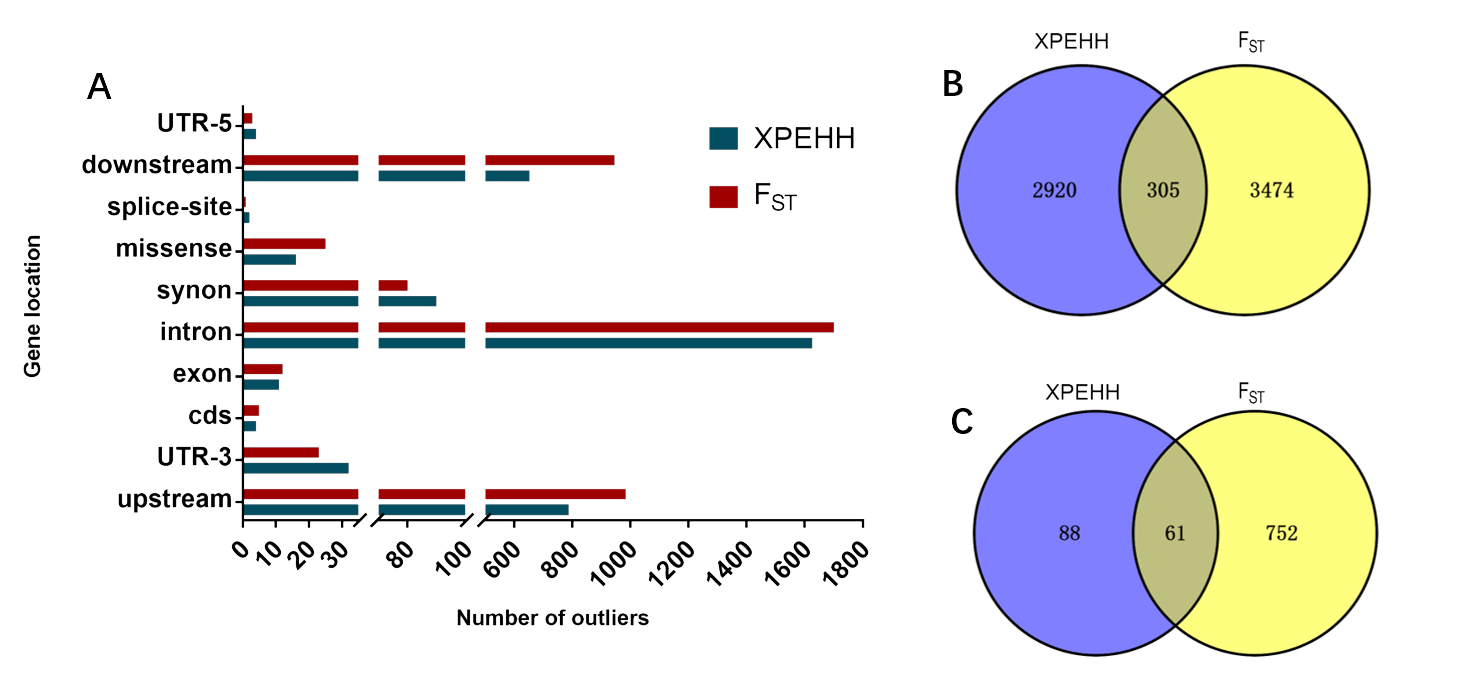

Supplement: Supplementary Figure 3 — Summary of outlier SNPs and genes from the identification of signature of selection. (A) Distribution of outliers in the gene structure detected by the FST and XP-EHH tests. Overlaps of outlier SNPs (B) and genes (C) detected by the FST and XP-EHH tests. [file Image_3.tif]

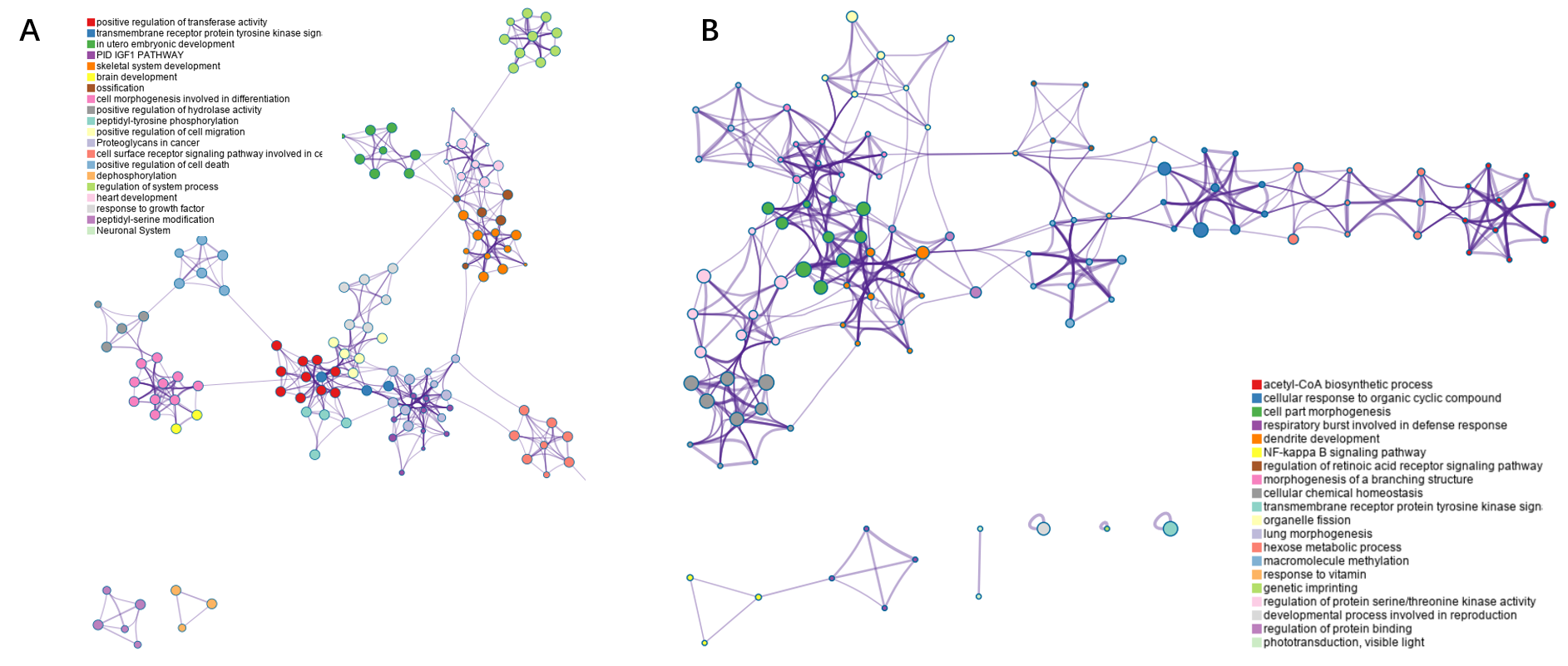

Supplement: Supplementary Figure 4 — Gene enrichment analysis. (A) The category clusters from XP-EHH test. (B) The category clusters from FST test. [file Image_4.tif]

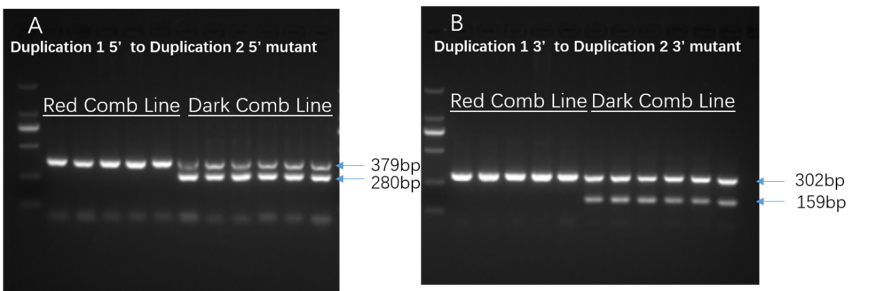

Supplement: Supplementary Figure 5 — Diagnostic test of boundaries of both the duplicated regions. The dark comb chickens displayed two bands in the agarose gel indicating the duplications in the genome, whereas the red comb chickens without the variant of genomic structure showed one band in the gel. [file Image_5.tif]
